# Surgical and functional outcomes and survival following Colon Cancer surgery in the aged: a study protocol for a prospective, observational multicentre study

**DOI:** 10.1186/s12885-021-08454-8

**Published:** 2021-06-14

**Authors:** Susanna Niemeläinen, Heini Huhtala, Anu Ehrlich, Jyrki Kössi, Esa Jämsen, Marja Hyöty

**Affiliations:** 1 Department of Gastroenterology and Alimentary Tract Surgery, Tampere University Hospital, Tays Hatanpää, P.O. Box 2000, 33521 Tampere, Finland; 2 Faculty of Social Sciences, Tampere University, Tampere, Finland; 3 Jorvi Hospital, Helsinki University Hospital, Helsinki, Finland; 4 Päijät-Häme Central Hospital, Lahti, Finland; 5 Tampere University Hospital, Centre of Geriatrics, Tampere, Finland; 6 Faculty of Medicine and Health Technology, Tampere University, Tampere, Finland; 7 Gerontology Research Center (GEREC), Tampere, Finland

**Keywords:** Colon cancer, Surgery, Aged patients, Functional ability, short-term outcome

## Abstract

**Background:**

The number of colorectal cancer patients increases with age. The decision to go through major surgery can be challenging for the aged patient and the surgeon because of the heterogeneity within the older population. Differences in preoperative physical and cognitive status can affect postoperative outcomes and functional recovery, and impact on patients’ quality of life.

**Methods / design:**

A prospective, observational, multicentre study including nine hospitals to analyse the impact of colon cancer surgery on functional ability, short-term outcomes (complications and mortality), and their predictors in patients aged ≥80 years. The catchment area of the study hospitals is 3.88 million people, representing 70% of the population of Finland. The data will be gathered from patient baseline characteristics, surgical interventional data, and pre- and postoperative patient-questionnaires, to an electronic database (REDCap) especially dedicated to the study.

**Discussion:**

This multicentre study provides information about colon cancer surgery’s operative and functional outcomes on older patients. A further aim is to find prognostic factors which could help to predict adverse outcomes of surgery.

**Trial registration:**

ClinicalTrials.gov (NCT03904121). Registered on 1 April 2019.

**Supplementary Information:**

The online version contains supplementary material available at 10.1186/s12885-021-08454-8.

## Introduction

### Background and rationale

The proportion of older people is increasing rapidly in western countries. In Finland, the estimated portion of the population aged over 70 will increase from the current 15.8 to 20% in 2030 and 25% in 2070 [1]. As the incidence of colon cancer increases with age [1, 2], the number of colon cancer patients can also be expected to increase, although the incidence has not dramatically changed over the decades [2].

The primary recommended treatment for colon cancer is surgery [3], but the decision to go through a major operation can be challenging for both the aged patient and the surgeon. Therefore, age-related concerns may lead to undertreatment of older patients, who compose a heterogeneous group with vastly different physiological and cognitive performance status [4]. Many older patients may have significant comorbidities, poor nutritional, and functional status [5, 6], which have been associated with severe postoperative complications associated with reduced 30-day and 1-year survival after colon cancer surgery [7]. Careful assessment is therefore required to make individual treatment decisions [8].

Modern medical and operative developments, including perioperative anaesthesia care, surgical performance (laparoscopy, technical standardisation), enhanced recovery program (ERAS) and oncological treatments, have improved outcomes for aged colorectal cancer patients [9, 10]. The difference in short-term postoperative mortality between older and younger colorectal patients has reduced during the last decade [11]. Conversely, frailty, a state of diminished physiological reserve capacity [12], has been identified as a significant predictor of postoperative complications leading to a prolonged hospital stay, discharge to nursing homes or long-term care facilities, and higher mortality rates than for fit patients [13, 14]. Preoperative screening tests for evaluating malnutrition, functional performance status, anaesthesiologist risks, and the cumulative burden of comorbidities can help identify factors that increase the risks of postoperative adverse events. However, comprehensive geriatric evaluations are often time-consuming and demand resources, highlighting the need for easily implemented screening tools, which produce crucial and objective results [15].

Older patients commonly prioritise functional outcomes before survival after cancer surgery [16]. Recent studies from the Netherlands and Norway reported positive impacts on quality of life after surgery for aged patients with colorectal cancer [17, 18]. There is growing evidence that physical and mental prehabilitation strategies, together with innovative surgical and postoperative treatments, can enhance long-term outcomes, functional recovery, and quality of life after surgery [19, 20]. A recently published international multicentre prospective study, Geriatric Oncology Surgical Assessment and Functional rEcovery after Surgery (GOSAFE), showed that 68.4% of patients over 70 years experiencing cancer surgery are frail. Thus, frailty evaluation has an essential role in predicting postoperative morbidity and mortality correlating with quality of life and physical and cognitive functional recovery [21].

There is little prospectively collected published data about the influence of colorectal surgery on postoperative outcomes and functional recovery for very old patients. The consensus recommendations and studies are mainly made for patients over 70 years [4, 8, 21]. Patients over 80 years are seldom included in prospective clinical trials, so the optimal treatment of these patients remains unclear [22]. In Finland, the incidence of colon cancer patients over 80 years has increased from 183 to 216 per 100,000 in the past 20 years, and 28% of patients were aged 80 years or more in 2018 [23]. Thus, it is significant to have adequate and trustworthy information about colon cancer surgery and its effect on postoperative morbidity, functional recovery, and survival. Recognition of frailty is essential to reduce adverse outcomes. Preoperative real-life clinical data can provide objective and helpful measures to the surgeons planning interventions for aged patients [24]. These instruments should accurately predict surgery’s adverse outcomes and be easy to implement, and thus able to guide comprehensive decision-making [25].

### Objectives

This multicentre study aims to analyse the impact of colon cancer surgery on patients over 80 years, their functional ability, the occurrence of complications, and mortality during the first postoperative year and highlight predictors of these adverse outcomes. We also aim to investigate non-operatively treated patients’ progress and subsequent functional ability and survival [26].

## Methods

### Study design and setting

The study is an observational, prospective, cohort, multicentre study of patients aged 80 years or older diagnosed with stage I-III colon cancer. The patients are treated either non-operatively or with curative resection or a palliative procedure. The participating hospitals are Helsinki University Hospital, Tampere University Hospital, Turku University Hospital, Central Finland Central Hospital, North Karelia Central Hospital, Päijät-Häme Central Hospital, Satakunta Central Hospital, South Ostrobothnia Central Hospital and Vaasa Central Hospital. The catchment area of the study hospitals is 3.88 million people (of whom 219,900 are aged 80 years or over), representing 70.4% of Finland’s population [1].

The public health care system almost exclusively performs the treatment of malignant diseases in Finland. All citizens have equal access to health care independent of social or insurance status. These study hospitals represent majority of Finnish hospitals operating colon cancer patients. Hence, the study provides a nationwide spectrum of operative management of colon cancer on the aged population. The study is independent of any industrial sponsorships.

### Participants

Patients aged 80 years or over with recently diagnosed stage I-III colon cancer will be assessed for suitability for inclusion. General practitioners or endoscopic units make most consultations of colon cancer patients, so the data includes only the patients referred to surgical units for operative treatment.

### Eligibility criteria (Fig. 1)

#### Inclusion criteria

- Stage I-III colon cancer
- Age 80 years or older at the time of recruitment
- The study’s information is approved and signed by the patient, or a legally authorised representative or family member if the patient’s cognitive status has declined.

**Fig. 1:**
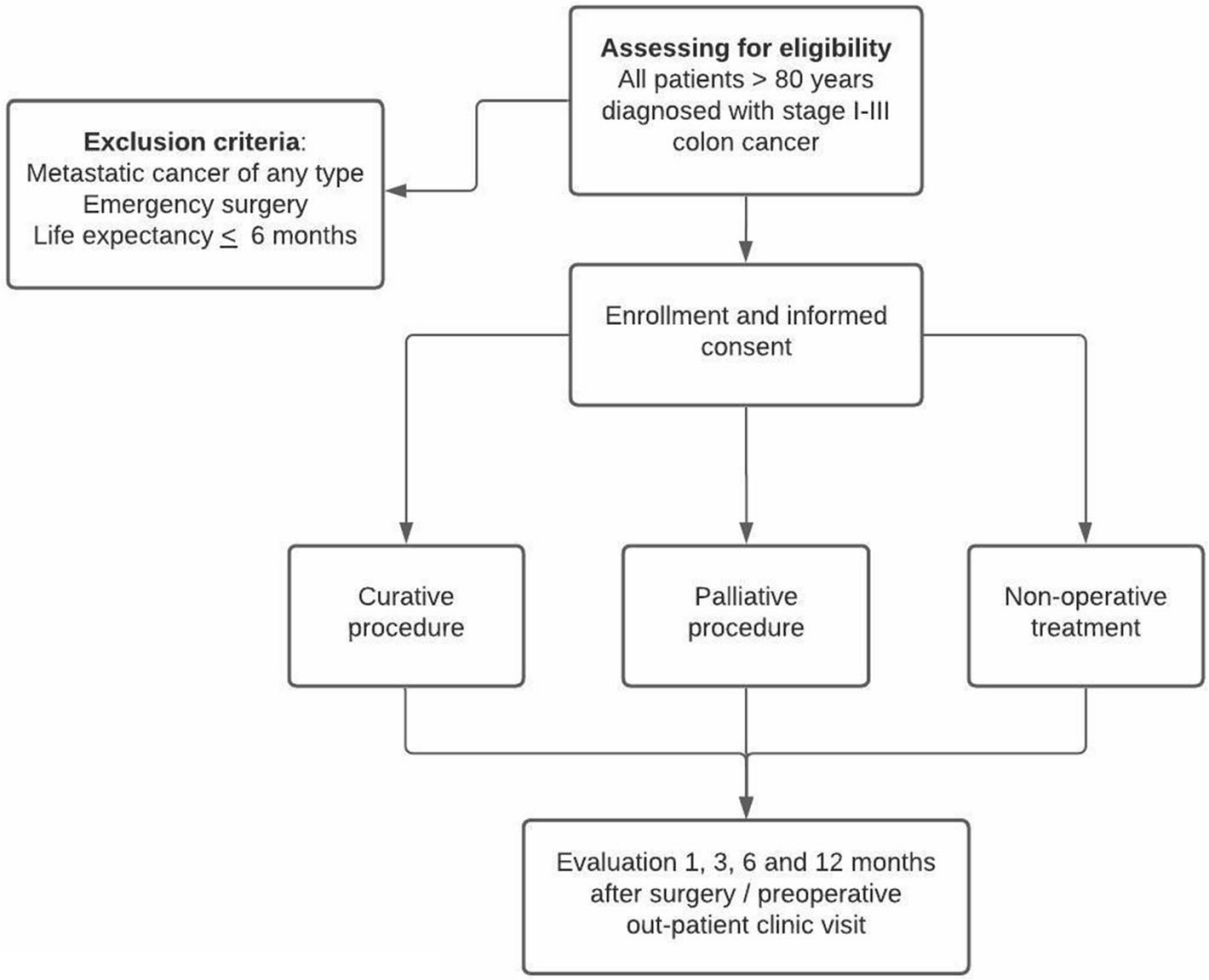
Eligibility criteria

#### Exclusion criteria

- Metastatic cancer of any type at the time of diagnosis with no possibility of curative surgery
- Patient undergoing emergency operation for colon cancer
- The patient has an expected life expectancy of less than 6 months due to colon cancer or other reasons.

### Primary intervention

All the patients have a preoperative colonoscopy, computed tomography, and presentation at a multidisciplinary team (MDT) meeting. They will undergo either radical surgery or a palliative procedure for the primary tumour, or nonoperative treatment according to hospital-standardised protocols for treatment and follow-up based on national EBM guidelines [27, 28]. The decision for definitive treatment choice is made collectively with the patient or, when needed, with a family member or legally authorised representative.

### Outcomes

The study’s primary outcomes are morbidity (surgical and non-surgical complications) and mortality, and their effect on functional recovery within 1 year after the primary surgical procedure, or after the date the decision for non-operative treatment is made. Postoperative morbidity will be recorded during the hospital stay, and at 30 days and 90 days after surgery, recorded at the outpatient clinic. Postoperative morbidity (surgical and non-surgical) will be assessed according to the Clavien-Dindo (CD) classification [29]. Mild complications are graded as CD I-II and severe complications as CD III-V. It will be recorded if patients with non-operative treatment require readmission to the study hospital. Mortality will be recorded at 30 days, 90 days and 1 year after surgery, or after the date that the decision for non-operative treatment is made.

Secondary outcomes are reoperations, length of hospital stay, discharge destination, readmission rate, and recruited patient (nursing home admission, mobility, and self-related health status [30]).

Long-term outcomes concerning functional recovery and mortality will be collected analogously 3 and 5 years after the cancer treatment decision.

### Tools for preoperative risk assessment

G-8: Onco-geriatric screening tool includes eight items modified from MNA-SF, age, number of medications and self-rated health status [31]. The G-8 score ranges from 0 to 17. Geriatric evaluation is recommended for patients whose score is ≤14 [32].

MNA-SF: Mini Nutritional Assessment-Short Form describes nutritional status as the normal, risk of malnutrition or malnourished. The score ranges from 0 to 14 and with cancer patients from 0 to 12. The risk of malnutrition is scored between 8 and 11 and malnourished patients between 0 and 7 [33].

CFS: Canadian Study on Health and Aging Clinical Frailty Scale, as assessed by the surgeon. The scale ranges from 1 to 9. Patients are described as frail with score ≥ 4 [34].

ASA: American Society of Anaesthesiologists physical status classification, which evaluates preoperative anaesthesiologist risk. The score ranges from 1 to 5. Categories are divided as 1 (a normal healthy patient), 2 (a patient with mild systemic disease, age > 65 years), 3 (a patient with severe systemic disease that is not incapacitating), 4 (a patient with an incapacitating systemic disease that is a constant threat to life), 5 (a dying patient who is not expected to survive for 24 h with or without operation) [35].

AA-CCI: Age-adjusted Charlson comorbidity index, which identifies cancer patients with increased risk of perioperative mortality. The scale ranges from 4 to 15; solid tumour excluded with two points, all patients 80 years or older contribute four points [36].

### Other information

Table 1 shows the information that is collected in addition to the risk assessment tools. The study surgeons record each patient’s baseline and data related to the surgery, perioperative hospitalisation, postoperative complications, and mortality. Patient-related questionnaires (Additional file 1) are collected before surgery, one, three, six and twelve months after surgery or the date non-operative treatment decision was made. The questionnaires will be collected either during the outpatient clinic visit, by telephone interview or by mail. All the information from the data mentioned above is recorded to the electronic database (REDCap) by the study surgeons. The dates and causes of death are obtained from the Death Certificate Register of Statistics Finland, which registers all deaths in Finland [37].

**Table 1:** Collected information

	Preoperative	Surgery	Hospital stay	1 month	3 months	6 months	12 months
**PRE-AND POST-INTERVENTION DATA**							
Age	x						
Sex	x						
Height and weight, BMI	x						
Medication	x			x	x	x	x
ASA classification	x						
Clinical Frailty Scale-index (CFS)	x						
Comorbidities (include diseases subsumed in modifield Charlson Comorbidity Index)	x						
Haemoglobin, creatine, estimated GFR, albumin	x						
History of smoking and alcohol-consumption	x			x	x	x	x
Other reported cancers excluding colon cancer	x						
Diagnostic procedures (colonoscopy, CT scan)	x						
Onco-geriatric screening tool (G-8)	x			x	x	x	x
Mini Nutritional Assessment- Short Form (MNA-SF)	x			x	x	x	x
Status of living	x			x	x	x	x
Mobility	x			x	x	x	x
Neuropsychological status	x			x	x	x	x
Weight loss and food intake	x			x	x	x	x
Self-related health status	x			x	x	x	x
Patient information and approval	x						
**INTERVENTION DATA**							
Date of operation		x					
Used surgical technique (laparoscopy, open, conversion)		x					
Curative or palliative operation		x					
Demand for stoma		x					
Operative time and blood lose		x					
**POSTOPERATIVE DATA**							
Surgical complications (anastomotic leakage, bleeding, ileus, wound dehiscence or infection, other) Clavien-Dindo classification		x	x	x	x		
Non-surgical complications (cardiovascular, pulmonary, urinary, delirium, other) Clavien-Dindo classification		x	x	x	x		
Reoperations			x	x	x		
Length of stay			x	x			
Place of discharge			x	x			
Readmissions			x	x	x		
Pathological report (TNM-status)				x			
Postoperative adjuvant treatment				x	x	x	
Recurrences (local and distant)				x	x	x	x
Date of death							

### Recruitment

All patients aged 80 years or older who have been diagnosed with stage I-III colon carcinoma and are referred to participating hospitals to consider surgery are eligible for the study. Surgeons responsible for the treatment will inform the patients about the possible advantages and disadvantages of the intervention and the study protocol of patient-questionnaires during their visit to the outpatient clinic before the definitive treatment. The information and consent forms are specially designed for the study and include the surgeons responsible for recruitment at each study site. After properly informed consent is obtained, the patient is recruited to the study.

Non-operative treatment is chosen if the patient is deemed unfit to survive the operation due to anaesthesiologic, physiological or cognitive status. If necessary, an anaesthesiologist, cardiologist, the pulmonary or geriatric specialist is consulted in decision-making. Patients may also refuse surgery after receiving information about possible advantages and disadvantages of the procedure. The ultimate decision will be made collectively with the patient and, if possible, with relations.

Both non-operatively and operatively treated patients are included, and they are not randomised in any way.

### Allocation

The study is an observational, prospective study without randomisation. All patients who voluntarily sign the consent form are included in the study. According to standardised protocols for treatment and follow-up, they will be treated at the study hospitals based on national EBM guidelines [27, 28]. The excluded patients are not followed up.

### Questionnaires used in the study

The pre-and postoperative questionnaires are specially designed for the study and are all convergent. The questions survey living status, use of implements, outside personal aid, functional and cognitive ability, and nutrition utilising G-8, MNA-SF and CFS [31, 33, 34]. They are collected before surgery and one, three, six and twelve months after surgery. The use of large font size (Calibri 16) aims to help these older people to answer the questions. The questionnaires are writing in Finnish or Swedish thus patients can use their native language.

The questionnaires (Additional file 1) are composed of following questions:

- status of living (home, nursing home, health centre wards)
- mobility (with or without implements, outside help)
- neuropsychological problems
- nutrition (food intake, weight-loss)
- self-rated health status in comparison with other people of the same age
- number of medications
- number of hospital admissions 6 months before the index surgery
- smoking habits and alcohol consumption
- living will (yes or no)

### Participant timeline

Recruitment started in April 2019 at Tampere University Hospital. The data collected from the participants and follow-up timeline is presented in Table 1. Short-term outcomes, including discharge history and complications, will be monitored at discharge, 1 and 3 months after primary operation by the surgeon responsible for recruitment.

### Sample size

The primary endpoints are the surgical and non-surgical postoperative complications and mortality. Previously published Finnish registry-based cohort studies on patients over 80 years with colorectal cancer reported complications rates 30–40% and severe complications 18–21%, respectively [7, 38]. In a large population-based cohort study with very old colorectal cancer patients, one-year mortality rates after surgery were 15–24% [39]. Frail patients had 2–3 times higher risk than non-frail patients of developing moderate to severe complications [14], leading to a disproportionately high risk of short-term mortality. A recently published study showed five times higher one-year mortality rate for patients with severe complications (8.6 vs 45%) [7].

Based on expected incidences of complications of 30% (fit patients) and 55% (frail patients), a sample size of 140 is needed to give 80% power to detect a significant difference between CFS 1–3 vs ≥ 4 groups, with two-sided type 1 error of 5%. Each group would comprise 70 patients. Additionally, with an estimated rate of one-year mortality of 10% (fit patients) and 25% (frail patients), we will need to study 113 frail subjects and 113 fit control subjects to be able to reject the null hypothesis that the failure rates for frail and fit subjects are equal with probability (power) 0.8. The type I error probability associated with this test of this null hypothesis is 0.05. We will use a continuity-corrected chi-squared statistic or Fisher’s exact test to evaluate this null hypothesis.

The first patient was included in the study 17th of April 2019 and operated on 29th April 2019. The preliminary estimate is that the final data will be collected by the end of April 2021.

### Data collection methods

#### Data management

An electronic database REDCap (https://www.project-redcap.org/) is used to gather the study data. REDCap is a secure web platform for building and managing online databases and surveys with online and offline data support. The main investigators have designed a dedicated version for this prospectively collected study data. All the surgeons responsible for the study at their hospital site receive a personal username and password for the electronic database to handle the data with confidentiality. Access to the whole database is limited to the main investigators in Tampere University Hospital and Tampere University.

All patients receive a study number, and the identification key is kept separately from the database on a username and password secured server at each study site. Data will be entered manually from paper case report forms (CRFs) into an electronic database (REDCap) protected by an automatic backup of server data and firewalls against external violation. All electronic case report forms (eCRFs) are handled with a particular study ID.

The occurrence of relevant protocol deviations such as metastatic disease, report of benign pathological tissue, or refusal to continue in the study will be determined and documented. Data verification and validation will be performed, and the results are analysed with code numbers not to identify the patient. When patient data and questionnaires have been coded, validated, and locked, a clean file will be declared.

#### Data collection

The principal investigator at each study site is responsible for the data collection and is reviewed by the main investigator. Each patient is asked to fill out patient-questionnaires at the time of inclusion, and at one, three, six and twelve months after surgery. If the patient cannot complete the questionnaire, a family member, legally authorised representative, or nurse in charge of the patient will complete the form. The principal investigators or research nurses of each study site are charged with ensuring that the patient questionnaires are completed.

Patients can resign from the study at any time during the study period, in this event data collected before resignation can be used in the analyses, following the last observation carried forward to practice.

#### Data monitoring

Instructions for data collection and storage have been provided to the surgeons responsible for recruitment at the study sites. The main investigator in Tampere University Hospital, who has full access to the full study register, will continuously monitor data. The other surgeons only have access to the patient register at their study site hospital. Technical and statistical monitoring, and advanced conduct with full access to the register, is given to the statistics expert from Tampere University.

#### Statistical methods

Percentages will be used to describe demographic data and the proportion of observed complications. The mean and standard deviation will be reported for age and the median and range for preoperative laboratory values and body mass index (BMI). Associations between the categorical variables are tested with the Chi-Square-test or the Fisher exact test, when appropriate. A uni- and multivariable analysis of the factors influencing morbidity and mortality will be carried out using binary logistic regression. All variables that were statistically significant in the univariate model are included in the multivariable model. Statistical analyses are performed using SPSS version 27.

### Ethics and dissemination

#### Research ethic approval

The study adheres to the Declaration of Helsinki on medical research protocols and ethics. Each participating hospital applies for study permission from the institutional review boards at their unit. The Regional Ethics Committee has approved the study protocol of the Expert Responsibility area of Tampere University Hospital (reference approval number R19028).

#### Protocol amendments

Significant protocol modifications are communicated with the Regional Ethics Committee of the Expert Responsibility area of Tampere University Hospital by amendments. All changes are also registered at ClinicalTrials.gov (NCT03904121).

#### Confidentiality

Patient confidentiality will be strictly maintained. Patients will be assigned a study ID, and all data will be handled without a name or personal social security number. Access to patient records is limited to the study group and the investigator-delegated study coordinator.

#### Dissemination policy

According to an agreement with the internationally accepted guidelines for authorship (International Committee of Medical Journal Editors), the study group members who are actively planning, recruiting, analysing, or writing will be part of the writing committee. Results will be published in peer-reviewed scientific journals. Results will also be communicated through professional meetings and the media.

## Discussion

With the increased life expectancy with the world population, the risk of developing colon cancer grows [2]. The decision to progress with invasive treatment can be challenging, as it should consider differences in preoperative physical and cognitive status that affect postoperative outcomes and functional recovery among the older population [19, 40]. Because of these differences, decision-making should not be based only on age. Instead, surgeons should evaluate the severity of comorbidities, functional and cognitive performance status to optimise a patient’s preoperative condition. Pre-operative risk assessments of postoperative outcomes, recognition of frailty, and identification of patients at greater risk of unfavourable treatment consequences, should be easy to implement.

This prospective, multicentre study will analyse colon cancer surgery’s impact on patients over 80 years, a patient group that will increase markedly in the coming years [1, 23]. The main objectives are short-term outcomes during the first postoperative year and their influences on functional ability and survival. These short-term outcomes are relevant as older colon cancer patients’ prognosis seems to be quite good (60% surviving at 5 years) if they avoid postoperative complications and survive the first postoperative year [41, 42].

Recent data from the GOSAFE study showed that 68.4% of patients were considered frail according to the G-8 score (≤14), and 36% had a cumulative burden of comorbidities (AA-CCI ≥7). In that study, 36.8% of patients were aged ≥80 years [21]. The present study will focus only on patients 80 years or older with potentially curable colon cancer. The very old express significant heterogeneity in physical and cognitive status, so we can expect frail patients with functional and cognitive impairment. In Finland, the life expectancy of an 80-year-old person is 8.3 years (male) and 10.2 years (female) [1], so it is essential to identify and evaluate prognostic factors, both favourable and adverse, which may predict how these patients recover from radical operative treatment. The collected data include readily available patient-related information about preoperative functional performance, preoperative examinations, the surgery, and the early postoperative course. Thereby, it allows analysis of patient- and surgery-related factors as predictors of early postoperative complications. Another advantage is that, unlike most previous studies, the study will also include non-operatively treated patients from the same hospitals, allowing evaluation of patient-selection and possible side-effects of non-operative treatment.

Although an observational study cannot answer whether the surgery is beneficial or not, performing a randomised trial in this patient group is not realistic. Instead, it is clinically more relevant to study outcomes in an observational setting with less selection bias and more relevance to real-life. This study’s strengths include examining a representative cohort, independent of social or insurance status, treated at several secondary and tertiary care hospitals instead of single-centre analysis. The multicentre nature of this study also allows for the timely collection of the data. It is acknowledged that the tests used (e.g. G-8, Clinical Frailty Scale, MNA-SF, CCI) represent screening tests, and more thorough geriatric evaluation would be needed for precise diagnoses. However, geriatric services are not widely available in surgical units at present. Evaluation of comprehensive geriatric assessment and preoperative optimisation protocols [43] in older colon cancer patients remains a question for later studies. The present results could, however, provide the basis for patient-selection in such later intervention studies.

This is the first prospective, observational, multicentre study of aged Finnish patients with non-metastatic colon cancer focusing on their treatment and its effects on postoperative outcomes and functional recovery. Finland follows uniform and standardised protocols for colon cancer treatment so that this study will provide realistic and novel information on aged patients postoperative functional recovery.

### Trial status

The trial recruitment started on 17 April 2019, and it is estimated to be complete by the end April 2021.

## Supplementary Information

**Additional file 1.**

## Data Availability

The datasets generated or analysed during the current study are not publicly available due to Finnish laws on privacy protection.

## Abbreviations

AA-CCI: Age-adjusted Charlson comorbidity index
ASA: American Society of Anaesthesiologists physical status classification
BMI: Body mass index
CFS: Clinical Frailty Scale
CRF: Case report forms
eCRF: Electronic case report form
EBM: Evidence-Based Medicine
ERAS: Enhanced recovery after surgery
G-8: Onco-geriatric screening tool
GOSAFE: Geriatric Oncology Surgical Assessment and Functional rEcovery after Surgery
ID: Identity documentation
MDT: Multidisciplinary team
MNA-SF: Mini Nutritional Assessment-Short Form
REDCap: Secure web application for electronic database

## References

[1] Statistics Finland. www.stat.fi/tup/statfin/index.html. Accessed 30 Oct 2020.

[2] Jafari MD, Jafari F, Halabi WJ, Nguyen VQ, Pigazzi A, Carmichael JC, Mills SD, Stamos MJ (2014). Colorectal cancer resections in the aging US population: a trend toward decreasing rates and improved outcomes. JAMA Surg.

[3] Van de Velde CJ, Boelens PG, Borras JM, Coebergh JW, Cervantes A, Blomqvist L (2014). EURECCA colorectal: Multidisciplinary management: European consensus conference colon & rectum. Eur J Cancer.

[4] Papamichael D, Audisio RA, Glimelius B, de Gramont A, Glynne-Jones R, Haller D, Köhne CH, Rostoft S, Lemmens V, Mitry E, Rutten H, Sargent D, Sastre J, Seymour M, Starling N, van Cutsem E, Aapro M (2015). Treatment of colorectal Cancer in older patients: International Society of Geriatric Oncology (SIOG) consensus recommendations. Ann Oncol.

[5] Barnett K, Mercer SW, Norbury M, Watt G, Wyke S, Guthrie B (2012). Epidemiology of multimorbidity and implications for health care, research, and medical education: a cross-sectional study. Lancet..

[6] Huisman MG, Veronese G, Audisio RA, Ugolini G, Montroni I, de Bock GH, van Leeuwen BL, Huisman MG, Veronese G, Audisio RA, Ugolini G, Montroni I, Vigano A, Gilbert L, Spiliotis J, Stabilini C, de Liguori Carino N, Farinella E, Stanojevic G, Veering BT, Reed MW, Somasundar PS, de Bock GH, van Leeuwen BL (2016). Poor nutritional status is associated with other geriatric domain impairments and adverse postoperative outcomes in Onco-geriatric surgical patients - a multi-Centre cohort study. Eur J Surg Oncol.

[7] Niemeläinen S, Huhtala H, Ehrlich A, Kössi J, Jämsen E, Hyöty M (2020). Risk factors of short-term survival in the aged in elective colon cancer surgery: a population-based study. Int J Color Dis.

[8] Montroni I, Ugolini G, Saur NM, Spinelli A, Rostoft S, Millan M, Wolthuis A, Daniels IR, Hompes R, Penna M, Fürst A, Papamichael D, Desai AM, Cascinu S, Gèrard JP, Myint AS, Lemmens VEPP, Berho M, Lawler M, de Liguori Carino N, Potenti F, Nanni O, Altini M, Beets G, Rutten H, Winchester D, Wexner SD, Audisio RA (2018). Personalized management of elderly patients with rectal cancer: expert recommendations of the European Society of Surgical Oncology, European Society of Coloproctology, International Society of Geriatric Oncology, and American College of Surgeons Commission on Cancer. Eur J Surg Oncol.

[9] Seishima R, Okabayashi K, Hasegawa H, Tsuruta M, Shigeta K, Matsui S, Yamada T, Kitagawa Y (2015). Is laparoscopic colorectal surgery beneficial for elderly patients? A systematic review and meta-analysis. J Gastrointest Surg.

[10] Bagnall NM, Malietzis G, Kennedy RH, Athanasiou T, Faiz O, Darzi A (2014). A systematic review of enhanced recovery care after colorectal surgery in elderly patients. Color Dis.

[11] Brouwer NPM, Heil TC, Olde Rikkert MGM, Lemmens VEPP, Rutten HJT, de Wilt JHW, van Erning FN (2019). The gap in postoperative outcome between older and younger patients with stage I-III colorectal cancer has been bridged: results from the Netherlands cancer registry. Eur J Cancer.

[12] Morley JE, Vellas B, van Kan GA, Anker SD, Bauer JM, Bernabei R (2013). Frailty consensus: a call for action. J Am Med Dir Assoc.

[13] Okabe H, Ohsaki T, Ogawa K, Ozaki N, Hayashi H, Akahoshi S, Ikuta Y, Ogata K, Baba H, Takamori H (2019). Frailty predicts severe postoperative complications after elective colorectal surgery. Am J Surg.

[14] Fagard K, Leonard S, Deschodt M, Devriendt E, Wolthuis A, Prenen H, Flamaing J, Milisen K, Wildiers H, Kenis C (2016). The impact of frailty on postoperative outcomes in individuals aged 65 and over undergoing elective surgery for colorectal Cancer: a systematic review. J Geriatr Oncol.

[15] Huisman MG, Kok M, de Bock GH, van Leeuwen BL (2017). Delivering tailored surgery to older Cancer patients: preoperative geriatric assessment domains and screening tools - a systematic review of systematic reviews. Eur J Surg Oncol.

[16] Akishita M, Ishii S, Kojima T, Kozaki K, Kuzuya M, Arai H, Arai H, Eto M, Takahashi R, Endo H, Horie S, Ezawa K, Kawai S, Takehisa Y, Mikami H, Takegawa S, Morita A, Kamata M, Ouchi Y, Toba K (2013). Priorities of health care outcomes for the elderly. J Am Med Dir Assoc.

[17] Souwer ETD, Oerlemans S, van de Poll-Franse LV, van Erning FN, van den Bos F, Schuijtemaker JS, van den Berkmortel FWPJ, ten Bokkel Huinink D, Hamaker ME, Dekker JWT, Wientjes CA, Portielje JEA, Maas HAA (2019). The impact of colorectal surgery on health-related quality of life in older functionally dependent patients with Cancer - a longitudinal follow-up study. J Geriatr Oncol..

[18] Rønning B, Wyller TB, Nesbakken A, Skovlund E, Jordhøy MS, Bakka A, Rostoft S (2016). Quality of life in older and frail patients after surgery for colorectal cancer—a follow-up study. J Geriatr Oncol..

[19] Ghignone F, Hernandez P, Mahmoud NN, Ugolini G (2020). Functional recovery in senior adults undergoing surgery for colorectal cancer: assessment tools and strategies to preserve functional status. Eur J Surg Oncol.

[20] Karlsson E, Egenvall M, Farahnak P, Bergenmar M, Nygren-Bonnier M, Franzén E, Rydwik E (2018). Better preoperative physical performance reduces the odds of complication severity and discharge to care facility after abdominal Cancer resection in people over the age of 70 - a prospective cohort study. Eur J Surg Oncol.

[21] Montroni I, Rostoft S, Spinelli A, Van Leeuwen BL, Ercolani G, Saur NM (2020). GOSAFE - geriatric oncology surgical assessment and functional rEcovery after surgery: early analysis on 977 patients. J Geriatr Oncol..

[22] Vermeer NCA, Claassen YHM, Derks MGM, Iversen LH, van Eycken E, Guren MG, Mroczkowski P, Martling A, Johansson R, Vandendael T, Wibe A, Moller B, Lippert H, Portielje JEA, Liefers GJ, Peeters KCMJ, van de Velde CJH, Bastiaannet E (2018). Treatment and survival of patients with Colon Cancer aged 80 years and older: a EURECCA international comparison. Oncologist..

[23] Finnish Cancer Registry. www.cancerregistry.fi. Accessed 30 Oct 2020.

[24] Amrock LG, Neuman MD, Lin HM, Deiner S (2014). Can routine preoperative data predict adverse outcomes in the elderly? Development and validation of a simple risk model incorporating a chart-derived frailty score. J Am Coll Surg.

[25] Huisman MG, Ghignone F, Ugolini G, Sidorenkov G, Montroni I, Vigano A, Liguori Carino N, Farinella E, Cirocchi R, Audisio RA, Bock GH, Leeuwen BL (2020). Long-term survival and risk of institutionalization in Onco-geriatric surgical patients: long-term results of the PREOP study. J Am Geriatr Soc.

[26] Abdel-Halim M, Wu H, Poustie M, Beveridge A, Scott N, Mitchell PJ (2019). Survival after non-resection of colorectal Cancer: the argument for including non-operatives in consultant outcome reporting in the UK. Ann R Coll Surg Engl.

[27] https://www.terveysportti.fi/dtk/ltk/koti?p_artikkeli=hsu00007. Accessed 30 Oct 2020.

[28] https://www.terveysportti.fi/apps/ltk/article/ykt00397/search/palliatiivinen%20hoito. Accessed 30 Oct 2020.

[29] Dindo D, Demartines N, Clavien PA (2004). Classification of surgical complications: a new proposal with evaluation in a cohort of 6336 patients and results of a survey. Ann Surg.

[30] Jylhä M (2009). What is self-rated health and why does it predict mortality? Towards a unified conceptual model. Soc Sci Med.

[31] Bellera CA, Rainfray M, Mathoulin-Pélissier S, Mertens C, Delva F, Fonck M, Soubeyran PL (2012). Screening older Cancer patients: first evaluation of the G-8 geriatric screening tool. Ann Oncol.

[32] Decoster L, Van Puyvelde K, Mohile S, Wedding U, Basso U, Colloca G (2015). Screening tools for multidimensional health problems warranting a geriatric assessment in older cancer patients: an update on SIOG recommendations. Ann Oncol.

[33] Rubenstein LZ, Harker JO, Salva A, Guigoz Y, Vellas B (2001). Screening for Undernutrition in geriatric practice: developing the short-form mini nutritional assessment (MNA-SF). J Geront.

[34] Rockwood K, Song X, MacKnight C, Bergman H, Hogan DB, McDowell I, Mitnitski A (2005). A global clinical measure of fitness and frailty in elderly people. CMAJ..

[35] Owens WD, Felts JA, Spitznagel EL (1978). ASA physical status classifications: a study of consistency of ratings. Anesthesiology..

[36] Chang CM, Yin WY, Wei CK, Wu CC, Su YC, Yu CH, Lee CC (2016). Adjusted age-adjusted Charlson comorbidity index score as a risk measure of perioperative mortality before Cancer surgery. PLoS One.

[37] Lahti RA, Penttilä A (2001). The validity of death certificates: routine validation of death certification and its effects on mortality statistics. Forensic Sci Int.

[38] Mäkelä JT, Klintrup KH, Rautio TT (2017). Mortality and survival after surgical treatment of colorectal cancer in patients aged over 80 years. Gastrointest Tumors.

[39] Claassen YHM, Bastiaannet E, van Eycken E, Van Damme N, Martling A, Johansson R (2019). Time trends of short-term mortality for octogenarians undergoing a colorectal resection in North Europe. Eur J Surg Oncol.

[40] Rostoft S, Hamaker ME (2020). Basic geriatric principles for colorectal surgeons: how to optimize assessment and Care of Older Patients in the perioperative period. Eur J Surg Oncol.

[41] Niemeläinen S, Huhtala H, Ehrlich A, Kössi J, Jämsen E, Hyöty M. Long-term survival following elective colon cancer surgery in the aged. A population-based cohort study. Colorectal Dis. 2020;9. 10.1111/codi.15242. 10.1111/codi.15242 32645253

[42] Mothes H, Bauschke A, Schuele S, Eigendorff E, Altendorf-Hofmann A, Settmacher U (2017). Surgery for colorectal cancer in elderly patients: how can we improve outcome?. J Cancer Res Clin Oncol.

[43] Partridge JLS, Harari D, Martin FC, Dhesi JK (2014). The impact of pre-operative comprehensive geriatric assessment on postoperative outcomes in older patients undergoing scheduled surgery: a systematic review. Anaesthesia.

