# Supplementary material for: Surgical and functional outcomes and survival following Colon Cancer surgery in the aged: a study protocol for a prospective, observational multicentre study
Source: BMC Cancer. 2021 Jun 14;21:698. doi: 10.1186/s12885-021-08454-8 (PMC8201898; doi:10.1186/s12885-021-08454-8)
Supplement: Supplementary file 1 — Additional file 1. [file 12885_2021_8454_MOESM1_ESM.docx]

**Additional file 1.** PREOPERATIVE AND POSTOPERATIVE SCREENING PATIENT-QUESTIONNAIRE

COLON CANCER SURGERY IN THE AGED, PROSPECTIVE OBSERVATIONAL STUDY

PREOPERATIVE / POSTOPERATIVE SCREENING PATIENT-QUESTIONNAIRE

| Date: |
| --- |
| Name: |
| Date of birth: |
| Sex:  □ Female  □ Male |
| Where do you live?  □ Home  □ Nursing home  □ Intensive residential care unit |
| Who do you live with?  □ Alone  □ With spouse or other family member  □ With somebody else |
| Do you need any help?  □ No  □ Yes, outside home  □ Yes, with housework  □ Yes, with every activity in daily life |
| How do you move?  □ Independently  □ Independently with implement  □ Only with assistance |
| How do you move outside home?  □ Alone  □ Yes, with assistance  □ I do not move outside |
| Implement for moving:  □ Wheelchair □ Walk frame  □ Cane  □ No assistance |
| In comparison with other people of the same age, how do you consider your health status?  □ Better  □ As good  □ Not as good  □ Do not know |
| Do you have dementia or depression?  □ No  □ Yes, I have mild dementia or depression  □ Yes, I have severe dementia or depression |
| Has your food intake declined over the past 3 months due to loss of appetite, digestive problems, chewing or swallowing difficulties?  □ Yes, severe decrease in food intake  □ Yes, moderate decrease in food intake  □ No  □ I cannot say |
| Have you had weight loss during the last 3 months?  □ Yes, weight loss > 3 kg  □ Yes, weight loss between 1-3 kg  □ No, I have not lost weight  □ Do not know |
| Have had hospital admissions 6 months before surgery?  □ No  □ Yes, once  □ Yes, several times |
| How many prescription drugs you take per day?  Number? |
| Do you use alcohol?  □ No  □ Yes, once a month or less frequently  □ Yes, once a week  □ Yes, several times a week |
| Do you smoke?  □ No  □ Yes |
| Do you have a living will?  □ No  □ Yes |
| Who filled out the form?  □ Patient  □ Relation  □ Nurse  □ Somebody else |
